# Practical Comparison of Two- and Three-Phase Bearingless Permanent Magnet Slice Motors for Blood Pumps

**DOI:** 10.3390/act13050179

**Published:** 2024-05-08

**Authors:** Jonathan E. M. Lawley, Giselle C. Matlis, Amy L. Throckmorton, Steven W. Day

**Affiliations:** 1 Departments of Biomedical and Mechanical Engineering, Kate Gleason College of Engineering, Rochester Institute of Technology, Rochester, NY 14623, USA; 2 BioCirc Research Laboratory, School of Biomedical Engineering, Science, and Health Systems, Drexel University, Philadelphia, PA 19104, USA; 3 Department of Pediatrics, College of Medicine, St. Christopher’s Hospital for Children, Drexel University, Philadelphia, PA 19104, USA

**Keywords:** bearingless, bearingless permanent magnet slice motor, FEA, two-phase, three-phase, rotary blood pump, mechanical circulatory support

## Abstract

The majority of bearingless permanent magnet slice motors (BPMSMs) used in commercially available rotary blood pumps use a two-phase configuration, but it is unclear as to whether or not a comparable three-phase configuration would offer a better performance. This study compares the performance of two-phase and three-phase BPMSM configurations. Initially, two nominal designs were manufactured and empirically tested for their performance characteristics, namely, the axial stiffness, radial stiffness, and current force. Subsequently, finite element analysis (FEA) models were developed based on these nominal devices and validated against the empirical results. Simulations were then employed to assess the sensitivity of performance characteristics to variations in seven different geometric features of the models for both configurations. Our findings indicate that the nominal three-phase design had a higher axial stiffness and radial stiffness, but resulted in a lower axial-to-radial-stiffness ratio when compared to the nominal two-phase design. Additionally, while the nominal two-phase design shows a higher current force, the nominal three-phase design proves to be slightly superior when the force generated is considered relative to the power usage. Notably, the three-phase configuration demonstrates a greater sensitivity to dimensional changes in the geometric features. We observed that alterations in the air gap and rotor length lead to the most significant variations in performance characteristics. Although most changes in specific geometric features entail equal tradeoffs, increasing the head protrusion positively influences the overall performance. Moreover, we illustrated the interdependent nature of the head height and rotor height on the performance characteristics. Overall, this study delineates the strengths and weaknesses of each configuration, while also providing general insights into the relationship between specific geometric features and performance characteristics of BPMSMs.

## 1. Introduction

Bearingless permanent magnet slice motors (BPMSMs) offer a distinct advantage over systems with conventional bearings by eliminating the physical contact between the rotor and stator. This absence of contact between moving parts results in minimal wear from friction, contributing to the growing popularity of BPMSMs in fluid pump applications. Across various industries, from chemical to biomedical, BPMSM pumps have gained prominence. In the biomedical sector, the BPMSM has found success in medical applications including mechanical circulatory support devices and rotary blood pumps [1–7].

More than 60 million patients globally suffer from heart failure (HF), a debilitating severity of heart disease that renders the heart muscle unable to effectively drive blood to the vital and end organs in the body [8–10]. The current treatment paradigm involves the administration of pharmacologic agents; this results in symptomatic improvement, but does not halt the progression to HF [11,12]. The shortage of donor organs and the further difficulty of donor–recipient size matching create hurdles for cardiac transplantation and extend patients’ waiting periods. To address these challenges, alternative treatment strategies are employed to provide bridge-to-transplant circulatory support in the form of a blood pump [13–15].

**Figure 1.**
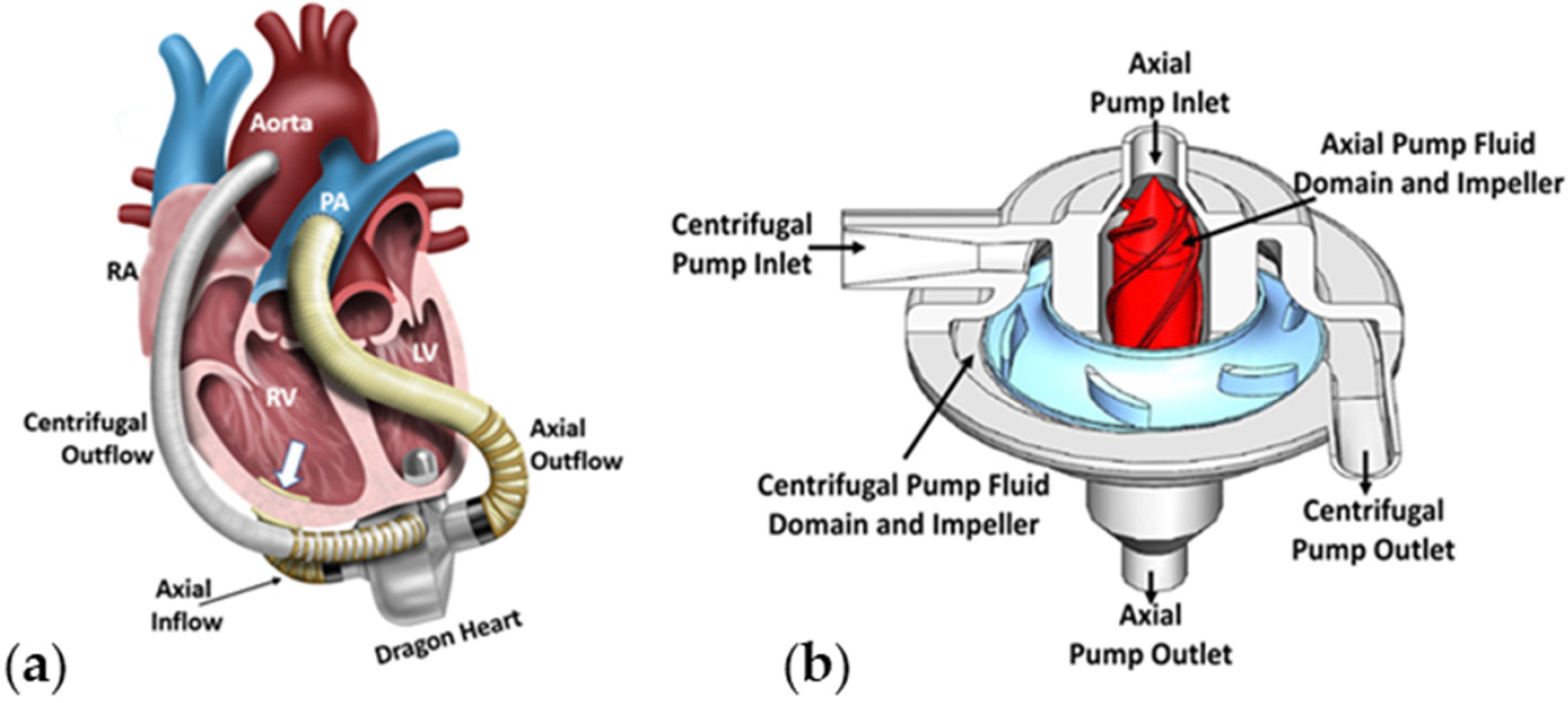
Continuous-flow, magnetically levitated Dragon Heart. (**a**) Implantation of the Dragon Heart medical device. The centrifugal blood pump is designed to support the systemic circulation and the left ventricle, and the axial flow blood pump is designed to support the pulmonary circulation and the right ventricle. RA: right atrium; RV: right ventricle; LV: left ventricle; PA: pulmonary artery. (**b**) TAH design details of the integrated axial and centrifugal pumps into a single device having only two moving parts, the levitated impellers [3].

Blood pumps are designed to supplement the output of the native left ventricle. These pumps generally operate in parallel with the beating diseased ventricle to provide adequate blood flow to the body. The design evolution of these medical devices has concentrated on the bearing support and motor drive systems [2–7]. First-generation blood pumps consist of pulsatile devices with pusher-plate or flexing diaphragms and valve configurations. Second-generation devices comprise continuous-flow or rotary pumps that require mechanical bearings and seals that are in contact with the fluid; and the latest generation of blood pumps, third-generation blood pumps, include axial and radial rotary pumps with no mechanical bearings in contact with the fluid medium, usually integrating magnetic or non-contacting hydrodynamic bearings [13,16]. Figure 1 illustrates a unique blood pump technology that integrates both an axial and a centrifugal pump into one medical device [3].

Notably, the two blood pumps most frequently used clinically, CentrigMag (Abbott, Abbott Park, IL, USA) and HeartMate III (Abbott, Abbott Park, IL, USA), share a two-phase configuration [17,18]. In contrast, BPMSMs in other industries conventionally employ three-phase motors due to the abundance of empirical and developmental data that are readily available. It has not been determined whether there are inherent benefits to one configuration over the other for medical applications such as blood pumps.

Three of the criteria that a designer should consider when choosing either a two-phase or three-phase configuration for a BPMSM blood pump are as follows: the size, air gap, and availability of off-the-shelf controller electronics. In the specific case of implantable blood pumps, there is the desire to reduce the size of the device in an effort to minimize the obtrusion to the user. For most BPMSMs, the quantity of phases will dictate the number of stator arms that are required, which will also affect the space between each arm. The size of this space is further limited by the sensors and coils that are present. Ultimately, there is a direct correlation between how small a device can be made and the quantity of phases used. Another important aspect of BPMSM blood pumps is that there are specific aspects that cannot be arbitrarily changed such as the air gap and blood gap. The air gap, also known as the magnetic gap, is the distance between the rotor and stator. The larger the air gap, the less influence the stator has on the rotor and, thus, the impeller. In a blood pump, a subset of the air gap is known as the blood gap which is defined as the distance between a wall of the impeller and a wall of the inner pump housing. It is advantageous for the blood gap to be large when compared to fluid gaps seen in more traditional pumps. The reason for this is because a small blood gap results in high shear within that region, which, in turn, could damage the blood [19]. The allowed blood gap is generally limited by the magnetic gap, which is, in turn, limited by the minimum allowable pump housing wall thickness. Thus, if either configuration could maintain sufficient control of the rotor with a larger air gap, then the allowable blood gap could be increased. The last thing a designer may consider is that off-the-shelf motor controllers are more prevalent for some phase configurations than others. The unique aspect of the BPMSM is that its rotor is magnetically levitated, but, aside from that, it simply functions as a traditional electric motor. Thus, the vast amounts of motor drive controllers made for traditional electric motors could be used with BPMSMs as long as they have the appropriate amount of phases.

Studies in the field of BPMSMs generally concentrate on developing novel topologies and control schemes [20–23]. Limited research has been carried out to determine the general relationship between specific geometric features and performance characteristics unique to BPMSMs. A notable exception is the study by Zhang et al. where they demonstrate the impact of three different geometric features on a configuration’s performance characteristics [24]. This study, however, only focuses on a two-phase configuration and does not offer a comparison to those with different amounts of phases. In studies where the number of phases is considered [25,26], the focus is predominantly on motor-driving characteristics such as the following: the acceleration, torque, and driving efficiency.

To address this knowledge gap, we investigated any innate difference between the two-phase and three-phase configurations and, thus, their appropriateness for use within blood pumps. We accomplished this by initially manufacturing nominal devices for the two-phase and three-phase configurations. The performance characteristics of axial stiffness, radial stiffness, and current force were empirically determined for these two manufactured devices. To extend the study beyond the comparison of only two designs, finite element analysis (FEA) was performed and allowed for dimensional changes for seven different geometric features. These simulations not only facilitated the comparison of how dimensional changes in geometric features affected the two configurations, but also, more broadly, enabled us to quantify the effect of critical geometric features on BPMSM performance. This provided valuable insights into the general nature of BPMSMs. Overall, the findings of this study provide a perspective on the advantages and disadvantages of two-phase and three-phase BPMSMs for use in blood pumps.

### 1.1. BPMSM Characteristics

**Figure 2.**
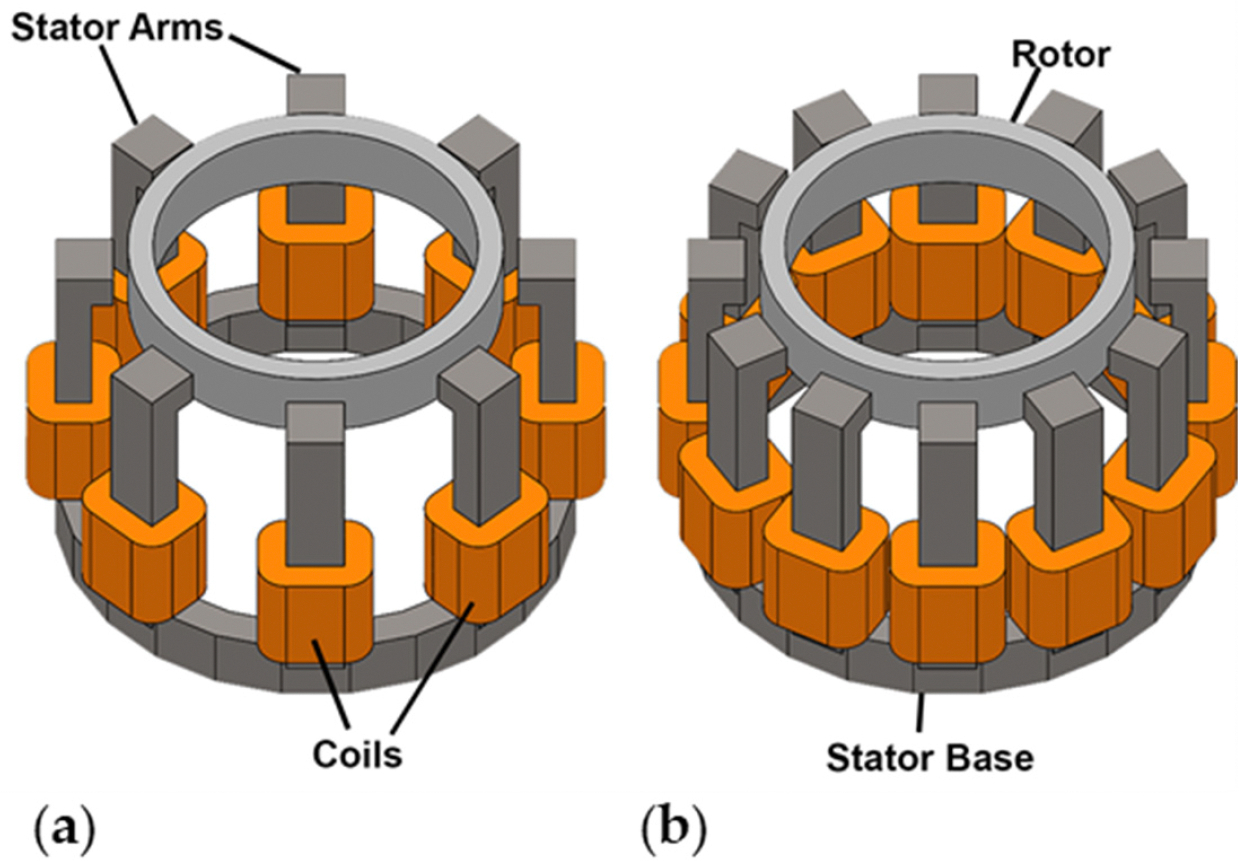
3D models of the two nominal designs with the pertinent components labelled. (**a**) The two-phase configuration and (**b**) the three-phase configuration. Note that, here, the rotors are displayed in their neutral position.

We evaluated the three critical performance characteristics that are unique to BPMSMs: the axial stiffness, radial stiffness, and current force. Figure 2 illustrates the nominal designs for the two-phase and three-phase configurations.

The axial and radial stiffness are passive characteristics that are a result of the interaction between the permanent magnet rotor and the ferrous stator. The passive characteristics are defined by the forces resulting from deviations from the rotor’s neutral position. We define the neutral position to be the location where the rotor is centered inside of the stator; in this neutral position, as shown in Figure 2, the axial and radial forces are zero. The axial stiffness (N/mm) is the force pulling the rotor towards the neutral position per axial distance displaced from the neutral position. Conversely, the radial stiffness (N/mm) is the force that pulls the rotor away from the neutral position per radial distance. In the majority of BPMSMs, it is operationally optimal to maintain the rotor’s position as close as possible to the neutral position. Another passive characteristic is the tilting stiffness which is the restoring torque per degree of the rotor tilt. The tilting stiffness is directly dependent on the axial stiffness for small tilt angles as a characteristic feature of BPMSMs is the large ratio of the rotor diameter to rotor height. This dependence is derived in Supplementary Materials. The axial stiffness and, as a result, tilting stiffness are both desirable due to their capability to return the rotor to its neutral position after an axial or tilt displacement, whereas the radial stiffness is undesirable because it can only displace the rotor from the neutral position. Then, there is the performance characteristic of the current force (N/A), which is the force acting on the rotor per current applied to the coils. The current force is an active characteristic because the user actively controls the force direction and magnitude. The purpose of the current force is to counteract the radial stiffness and center the rotor. Here, we define the current force as the radial force on the rotor per current. It is important to note that the radial stiffness and current force both depend on the rotor angle. To capture this, we analyzed and reported the 0° case as this corresponds with the maximum radial stiffness and current force. Lastly, another important active characteristic for BPMSMs is the motor drive performance, but this has been analyzed in prior work [25,26].

### 1.2. Suspension Principle

**Figure 3.**
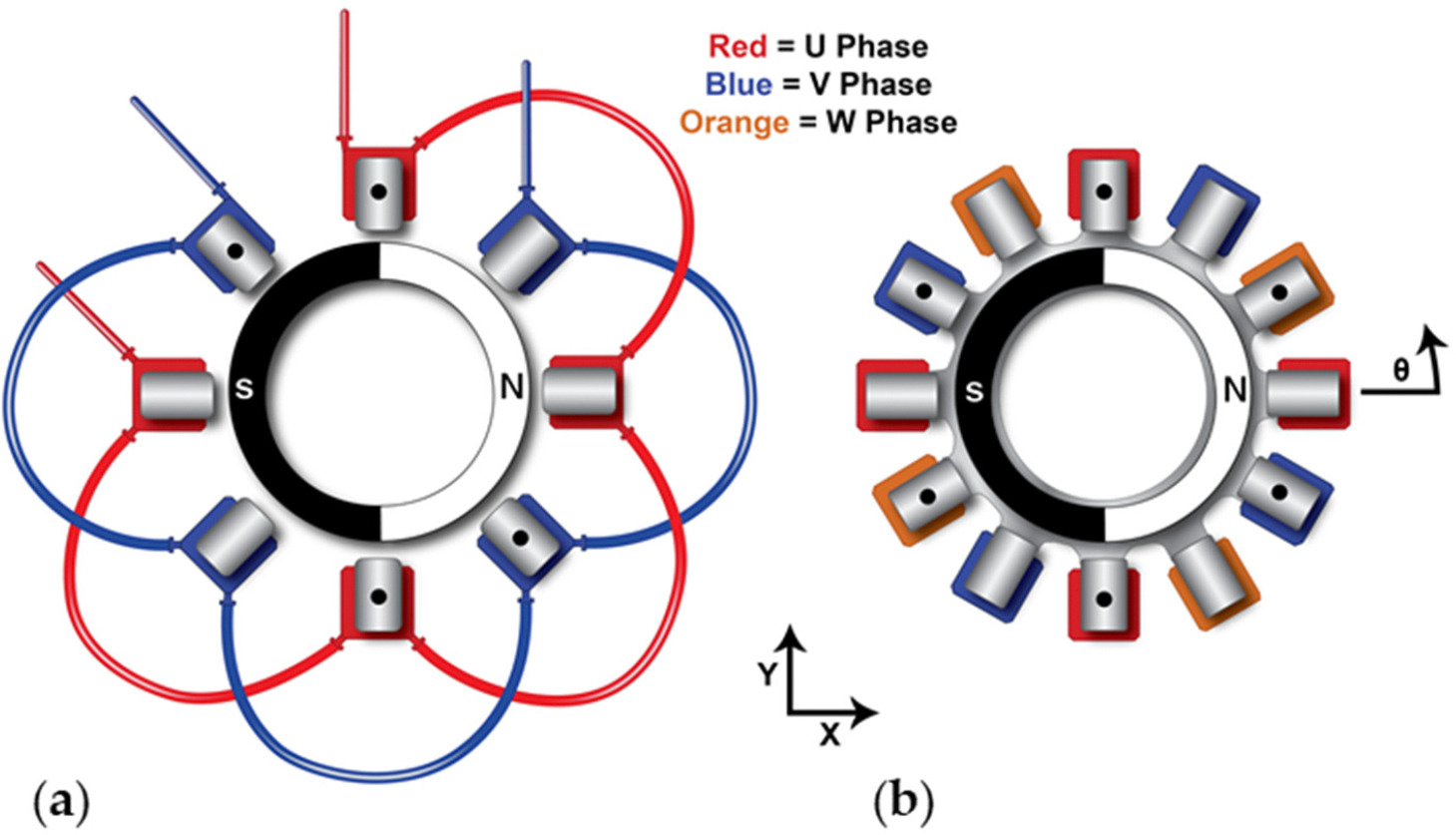
The two-phase and three-phase suspension wiring schemes when viewed from above. (**a**) Two-phase design with the wiring of the phases in series. (**b**) Three-phase design where the phases were wired similarly. The use of a black dot indicates that the coil was specifically wound counterclockwise; otherwise, the coil was wound in the clockwise direction.

While there are a wide variety of different suspension principles for two-phase and three-phase configurations, as reported by [27,28], we chose the standard $N_{ps}=N_{pr}+1$, where $N_{ps}$ represents the number of suspension pole pairs and $N_{pr}$ signifies the number of rotor pole pairs. The aforementioned suspension principle is commonly used [21,22] and corresponds to the configurations of both the CentriMag and HeartMate III [17,18]. Furthermore, the equal, conventional dipole rotor [29,30] was used in this study, meaning that $N_{pr}=1$, which then required that $N_{ps}=2$. As a result, the two-phase and three-phase configurations require 8 and 12 arms, respectively. Aside from these differences in the number of arms, the motors also have differing phase configurations, as seen in Figure 3.

As a result of different phase configurations, the current distributions for the active suspension, according to the rotor angle, are shown in Equations (1) and (2):

(1) $$I_{U}=\frac{I_{\mathrm{in}}\cos(\theta )}{2},I_{V}=\frac{I_{\mathrm{in}}cos\left(\theta \;+\;120^{\circ }\right)}{2},I_{W}=\frac{I_{\mathrm{in}}cos\left(\theta \;-\;120^{\circ }\right)}{2}$$

and

(2) $$I_{U}=I_{in}cos(\theta ),I_{V}=I_{in}cos\left(\theta +45^{\circ }\right)$$

where $I_{in}$ reflects the current input and θ is the rotor’s angle in degrees. These equations are defined such that the total amount of current in either equation would be identical at the same rotor angle.

## 2. Materials and Methods

To begin our analysis of two-phase and three-phase configurations, we started by selecting a single design for each configuration, which we refer to as the nominal designs. The manner in which the nominal designs were selected was by first selecting a general design and size that was able to facilitate either 8 or 12 arms. The models of these designs can be seen in Figure 2. The topology was a standard temple design commonly present in BPMSMs [29]. We opted to use a common base size and shape for the stator in both designs, varying only the number of grooves for the arms. Additionally, the arms themselves were identical between the two-phase and three-phase designs with the only difference being the number of arms.

The stator was constructed in-house and made from low-carbon steel 1018. The base and the arms were constructed separately, and then attached via bolts. Each arm received its own coil, which consisted of 150 windings of 24 AWG enamel wire. For the rotor, we employed a custom-grade N50 NdFeB permanent ring magnet with diametric magnetization (SM Magnetics, Pelham, AL, USA). Once the two nominal devices were manufactured, they were then tested to empirically evaluate their performance characteristics.

Afterwards, FEA models of the two nominal designs were created using COMSOL Multiphysics (v. 5.2, COMSOL AB, Stockholm, Sweden), which were informed by results of the empirical tests. The models replicated the manufactured device in both dimensions and materials to the best of our ability. FEA was employed to facilitate a more complete comparison of the two-phase and three-phase configurations by allowing the dimensions to be varied for seven geometric features without requiring different designs to be manufactured.

### 2.1. Experiments

**Figure 4.**
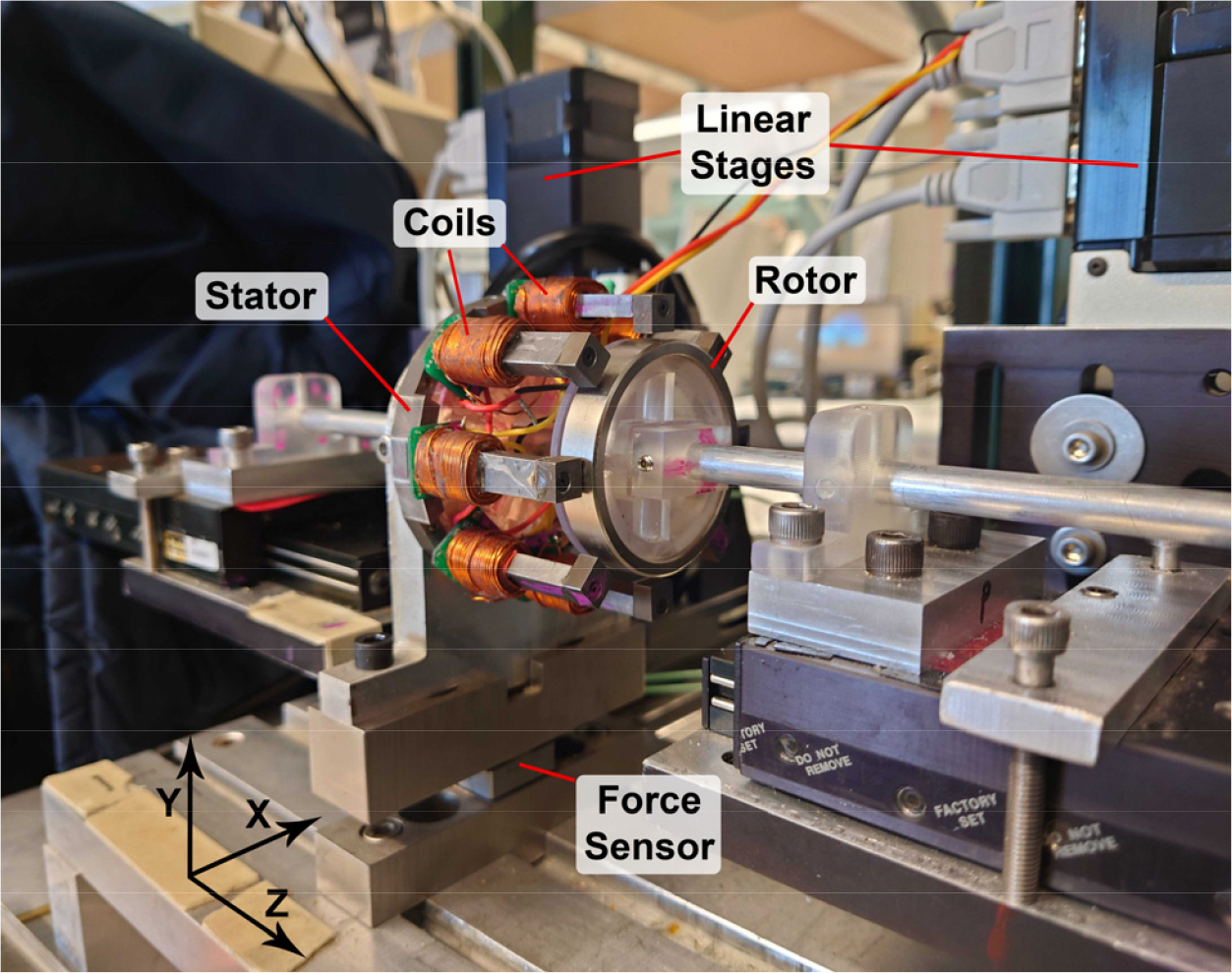
Two-phase nominal design positioned on the force testing rig. Rotor at the centered, neutral position. The rotor and linear stages are moveable, whereas the coils, stator, and force sensor remain stationary.

Figure 4 illustrates a custom test rig that was used to measure the BPMSMs’ axial stiffness, radial stiffness, and current force. A schematic representation of the test rig’s components is shown in Supplementary Materials Figure S3. The forces were measured in each nominal design by attaching the stator to a three-component force sensor (Kistler Instrument Corp, 9251A, Amherst, NY, USA) via a custom aluminum fixture. The rotor was attached to an aluminum rod via a custom 3D-printed piece. The rod was then rigidly affixed to two separate three-axis translation stages that allowed for its precise positioning for the aforementioned rotor within three-dimensional space. Each stator phase coil grouping was wired to an individual operational amplifier (Apex Microtechnology Inc., PA02A, Tucson, AZ, USA) which allowed for exact allocation of current. Control of the linear stages, current distribution, and data acquisition from the load cell were accomplished using a custom LabVIEW (v. 18.0.1f4) code. The initial rotor position for each experiment was defined as the point where the rotor is centered axially and radially with a 0° rotor angle. To determine the axial stiffness, the rotor was shifted to five evenly spaced axial positions from the points −2 mm to +2 mm and the force was recorded at each location. For radial stiffness, the rotor was moved to five evenly spaced radial positions along the x-axis from the points −1 mm to +1 mm. Finally, to measure the current force, the amperages of 1A, 2 A, 3A, 4A, and 5A were applied to the specific phases, in accordance with Equations (1) and (2).

### 2.2. Simulations

**Figure 5.**
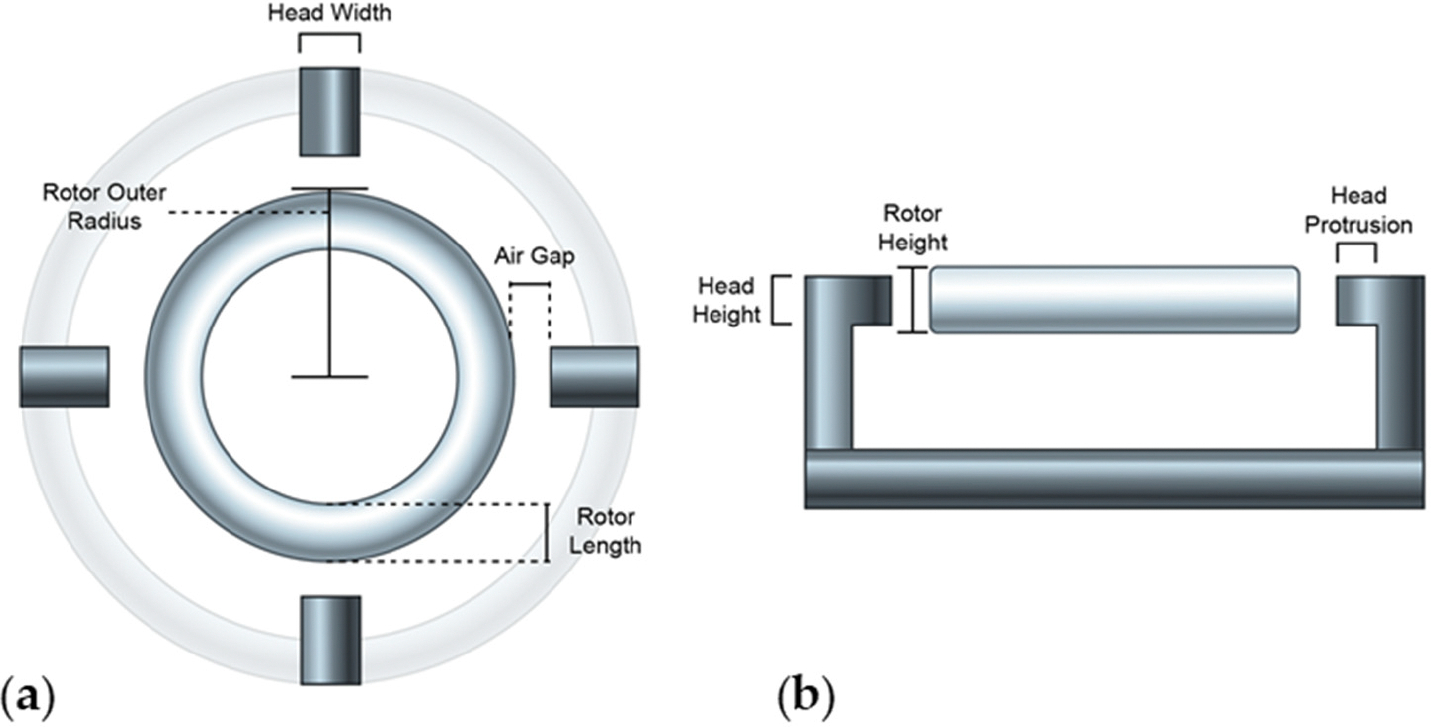
Illustration of the seven key geometric features of the two-phase and three-phase BPMSMs. (**a**) Rotor outer radius, head width, air gap, and rotor length are shown; top view. (**b**) Head height, rotor height, and head protrusion are shown; side view.

After empirical testing was completed, we created FEA models of the nominal designs using COMSOL Multiphysics. As mentioned previously, these models replicated the materials and design of the manufactured nominal devices. We then determined the axial stiffness, radial stiffness, and current force by using displacements and currents identical to the ones used in the empirical studies. Validation of the models was assessed by determining the difference between the simulated and empirical results. Our models allowed for the varying of dimensions of seven different geometric features and enabled the evaluation of their effect on the performance of the two different configurations. For this study, we chose to investigate the seven geometric features as follows: air gap, head height, head protrusion, head width, rotor height, rotor length, and rotor outer radius; they are detailed in Figure 5. There are other geometric features including, but not limited to, the stator arm height and baseplate height, that may have an effect on the BPMSMs’ performance characteristics. We prioritize the seven geometric features that we selected based on their proximity to the air gap.

Along with the nominal design, two other dimensions were tested for each geometric feature as illustrated by Table 1. For this study, only the dimension of a single geometric feature was varied from the nominal design at a time. The two additional dimensions that were selected for each geometric feature were based upon realistic design extremes such as the concept that geometric features cannot be arbitrarily reduced and maintain structural integrity. The values of these dimensions are further limited by physical constraints of the nominal designs. The considered dimensions enabled the sensitivity to be determined for the performance characteristics in response to changes in geometric features. Comparisons were made by calculating the percent difference between the two nominal simulated results. Lastly, preliminary results suggested an interdependent relationship between the geometric features of head height and rotor height. Thus, we simulated all combinations of the dimensions listed in Table 1 for the head height and rotor height.

## 3. Results and Discussion

### 3.1. Numerical and Empirical Agreement

Table 2 details the performance characteristics for the empirical studies and simulations of the nominal designs. Supplementary Materials Figure S4 demonstrates all of the comparisons between the numerical and empirical tests that were used to derive the aforementioned performance characteristics. The discrepancy between the empirical studies and simulations ranged between 10.3–21.0% for all cases. One source of discrepancy is the fact that the materials used in simulation are the idealized versions whereas inconsistencies in the manufacturing process of these same materials can lead to differing magnetic properties. Additionally, the engineering tolerances of the manufactured device can affect the difference between the numerical and empirical results. This is especially true for tolerances that would affect the air gap because, as we will discuss in later sections, small changes in the air gap have a large impact on the performance characteristics, particularly with regard to the radial stiffness.

There has been little precedent to determine what defines an adequate agreement between the numerical and empirical data specifically in the case of BPMSMs. In [31], they studied a design similar to our three-phase configuration and they reported 13–38% discrepancies for the performance characteristics of the current force and radial stiffness. With that said, it is important to note that, despite the discrepancies, the trends for both empirical and numerical data as seen in Supplementary Materials Figure S4 are similar in the sense that they are both linear. The reason this aspect of the data is highlighted is because it demonstrates that the underlying mechanisms of the FEA model are in line with those of the physical devices. This is corroborated by the fact that the percent difference of the numerical and empirical results for the three performance characteristics are consistent between the two-phase and three-phase nominal designs. What this means is that, while there are discrepancies within the absolute values, changes in the dimensions of geometric features in the simulations would consistently be 10.3–21.0% different from their empirical counterpart. This then bolsters our FEA model’s usage in the simulations of this study, the purpose of the simulation being to show the effect of dimensional changes in geometric features between the two-phase and three-phase configurations.

### 3.2. Nominal Comparisons

**Figure 6.**
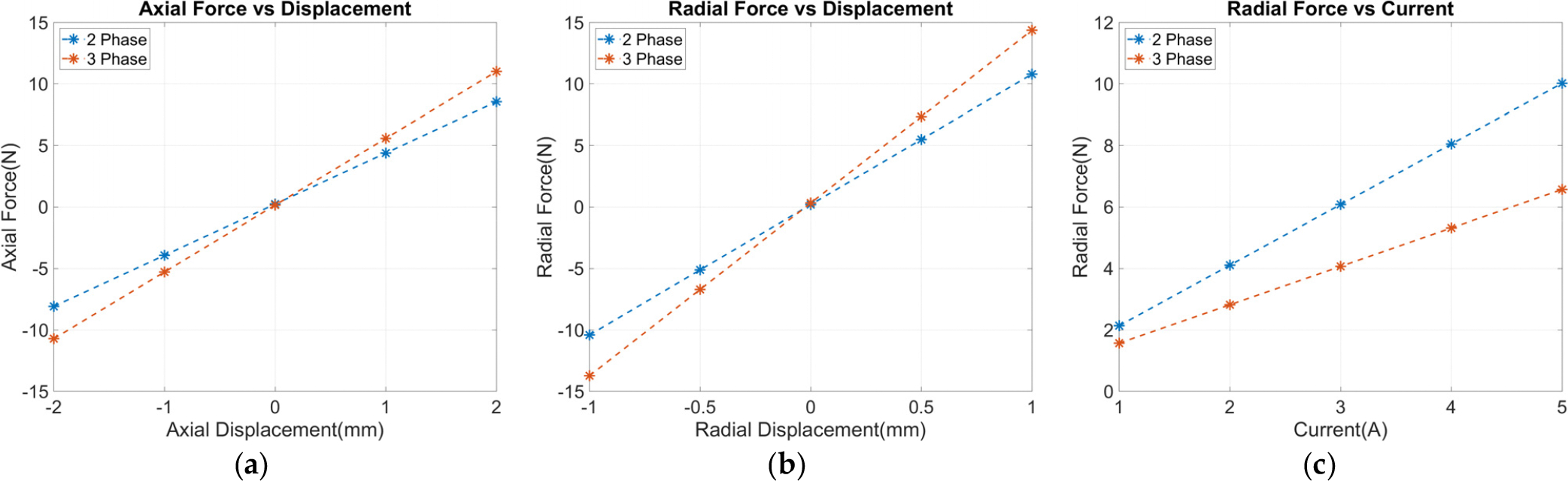
Comparison of the two-phase and three-phase nominal designs: (**a**) the axial force as a function of axial displacement (slope is axial stiffness (N/mm)); (**b**) radial force as a function of radial displacement (slope is radial stiffness (N/mm)); and (**c**) the radial force as a function of current (slope is current force (N/A)).

Figure 6 illustrates the experimental results for the axial stiffness, radial stiffness, and current force for the nominal designs. This figure demonstrates the linearity of these three functions within the range of independent values that we explored. Table 3 succinctly summarizes these performance characteristics and provides a quantitative comparison between the two nominal designs.

The three-phase nominal design demonstrated higher axial and radial stiffnesses, which can be attributed to the fact that the magnetic force is proportional to the surface area [28]; the three-phase design has more arms and, therefore, a greater surface area for a given arm design than the two-phase design. We propose that the ratio of the advantageous axial stiffness to the detrimental radial stiffness can be used to evaluate the general performance of any BPMSM design. In this case, we find that the three-phase nominal design has a lower axial-to-radial-stiffness ratio, 0.386 as compared to 0.392.

**Figure 7.**
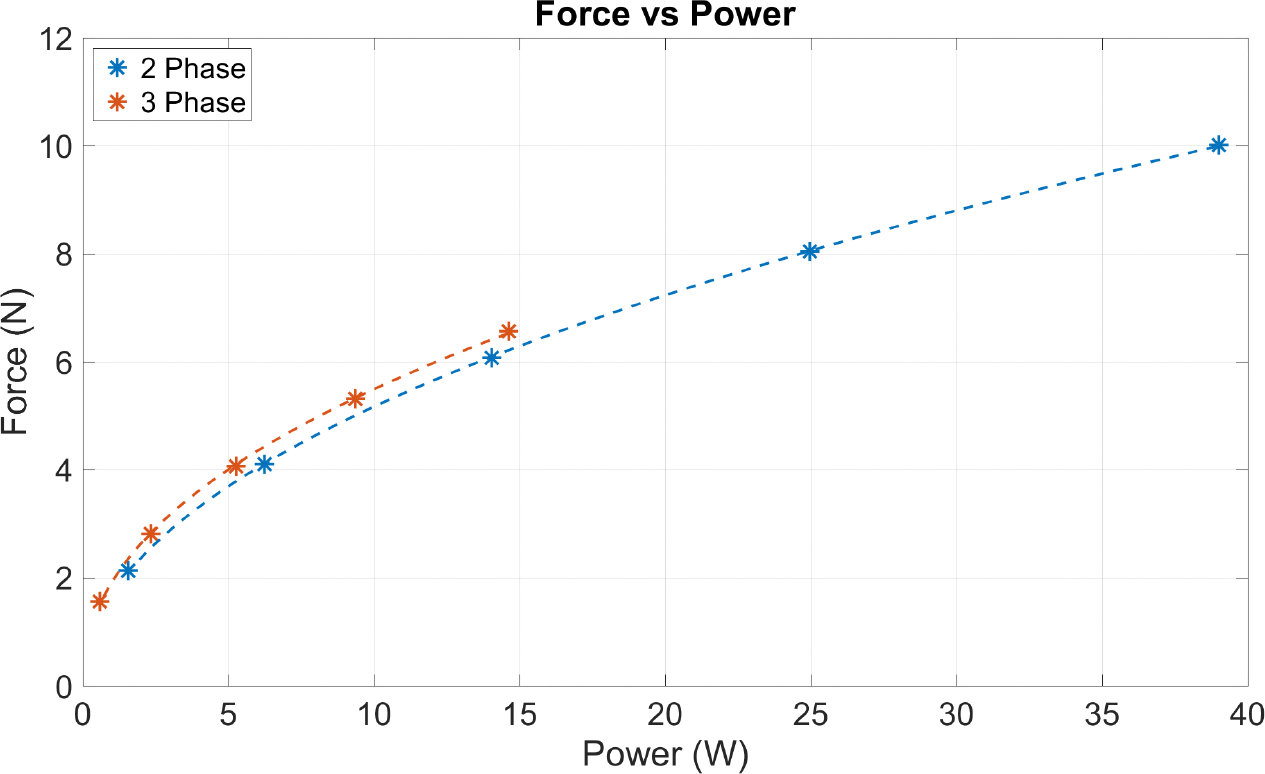
Force generation as a function of power for the nominal two-phase and three-phase designs.

The two-phase motor produces a substantially higher (45.4%) current force than the three-phase designs. This is due to the differing distribution of the total current as per Equations (1) and (2). Thus, the total power usage of the three-phase design will be lower than the two-phase design. Figure 7 displays the current force as a function of power rather than current.

Per Figure 7, more similar efficiencies between the two-phase and three-phase configurations were observed when the force was represented as a function of power, rather than a function of current; Figure 6c. The relationship demonstrated in Figure 7 can then be represented as the following function:

(3) $$F=k\sqrt{P}$$

where F is the force generated (N), P is the power usage (W), and k is the proportionality constant (N/W). k for the two-phase design is 1.57 N/W and that for the three-phase design is 1.62 N/W, which is a 2.7% difference. At this point, it is important to note that the purpose of the current force is to counteract the force resulting from the radial displacement. Thus, while similar force per power values were found, a radial displacement for the three-phase design requires more power than the two-phase design. As an example, a radial displacement of 0.5 mm requires 11.3 W and 18.8 W for the two-phase and three-phase nominal designs, respectively.

### 3.3. Sensitivity

**Figure 8.**
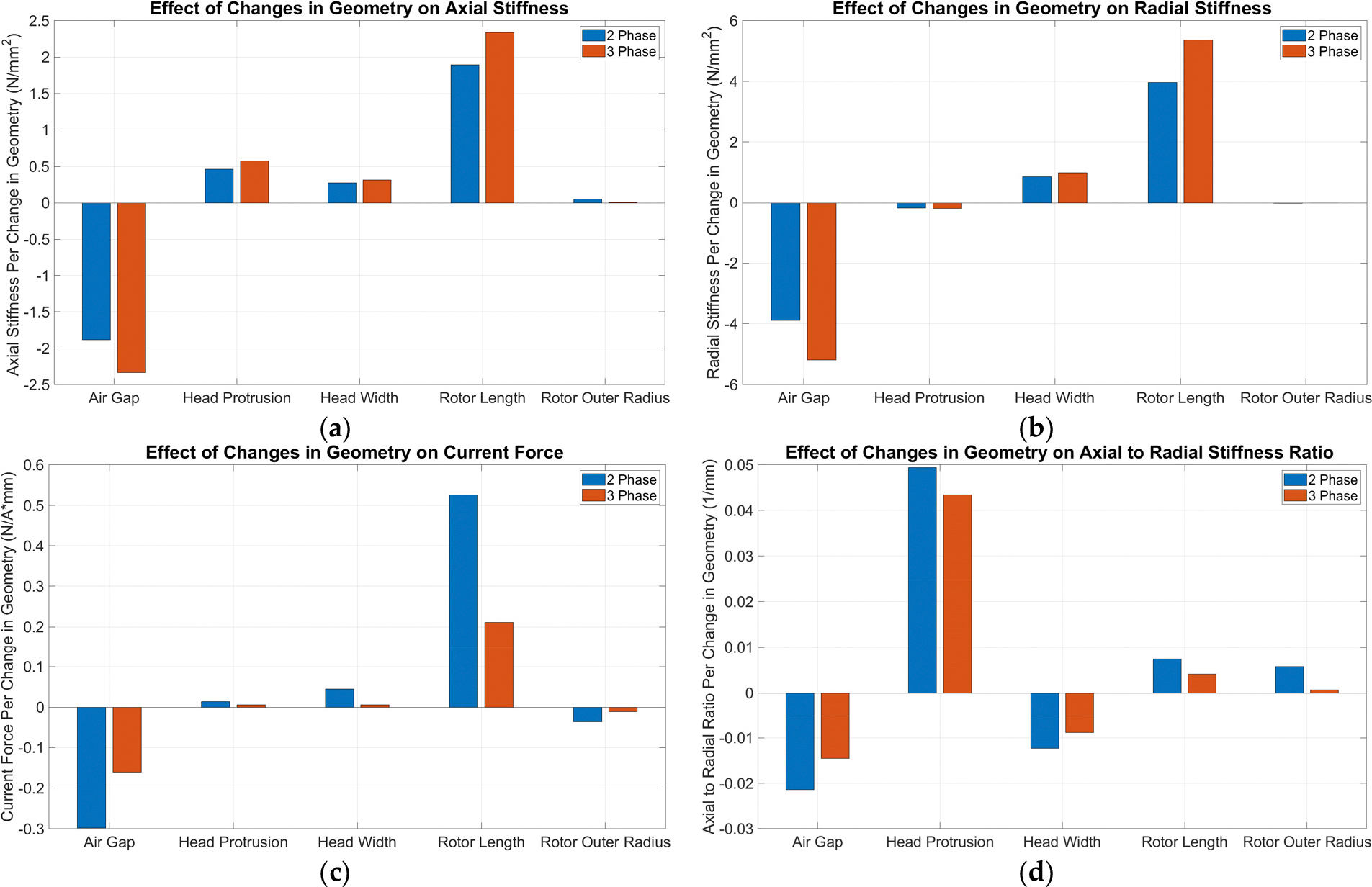
Sensitivity of (**a**) axial stiffness, (**b**) radial stiffness, (**c**) current force, and (**d**) axial-to-radial-stiffness ratio to changes in specified geometric features. Additionally, the comparison between the two-phase and three-phase configurations.

The purpose of using simulations to vary the dimensions of specific geometric features was twofold. The first reason was to find the sensitivity of the characteristics of each design to dimensional changes in these geometric features for both phase configurations. More generally, it allowed us to determine the relationships between specific geometric features and the active and passive characteristics of a BPMSM with this topology regardless of the configuration. For the ranges of dimensions that we chose to simulate, the relationship between the majority of the characteristics and changes in geometric features were linear, which allows us to represent the sensitivity for these geometric features as a single value as shown in Figure 8.

The results in Figure 8 represent how the characteristics of the nominal design would change per dimensional change of the specified geometric feature. Furthermore, a positive value means that increasing the dimension of that geometric feature would increase that performance characteristic, whereas a negative value reflects the opposite. Values of the sensitivities displayed in Figure 8, as well as the percent difference between the two-phase and three-phase configurations, are shown in Supplementary Materials Table S1. Additionally, the graphs from which Figure 8 is derived are shown in Supplementary Materials Figure S5.

In line with the performance characteristics of the nominal designs, we observe that the three-phase design has a higher sensitivity than the two-phase design for both passive characteristics as seen in Figure 8a,b. For example, the radial stiffness sensitivity for the rotor length was 5.4 N/mm^2^ versus 4.0 N/mm^2^ for three-phase design and two-phase design, respectively. Conversely, Figure 8d demonstrates a consistently higher axial to radial sensitivity for the two-phase configuration as can be seen by the air gap sensitivity being −0.021 1/mm and −0.014 1/mm for the two-phase and three-phase configurations, respectively. Lastly, the three-phase configuration had a lower sensitivity for the active characteristic as exemplified by its 0.21 NA/mm^2^ current force sensitivity to the rotor length as compared to the two-phase configuration’s 0.52 NA/mm^2^

Regardless of the configuration, we determine and present the general trends of the BPMSMs. It is shown that the rotor outer radius has virtually no effect on any of the characteristics. For example, the rotor outer radius’ axial stiffness sensitivities were only 0.05 N/mm^2^ and 0.09 N/mm^2^ for the two-phase and three-phase configurations, respectively. While most geometric features have the same sign for all of the sensitivities, the head protrusion does not; this suggests that increasing the head protrusion is always beneficial. Additionally, we found that all performance characteristics are sensitive to changes in the air gap and rotor length when compared to the other geometric features. As an example of this, the axial stiffness sensitivity for the two-phase configuration is −1.88 N/mm^2^ and 1.89 N/mm^2^ for the air gap and magnet length, respectively, but are only 0.46 N/mm^2^ and 0.27 N/mm^2^ for the head protrusion and head width, respectively.

### 3.4. Interdependent Geometric Features

**Figure 9.**
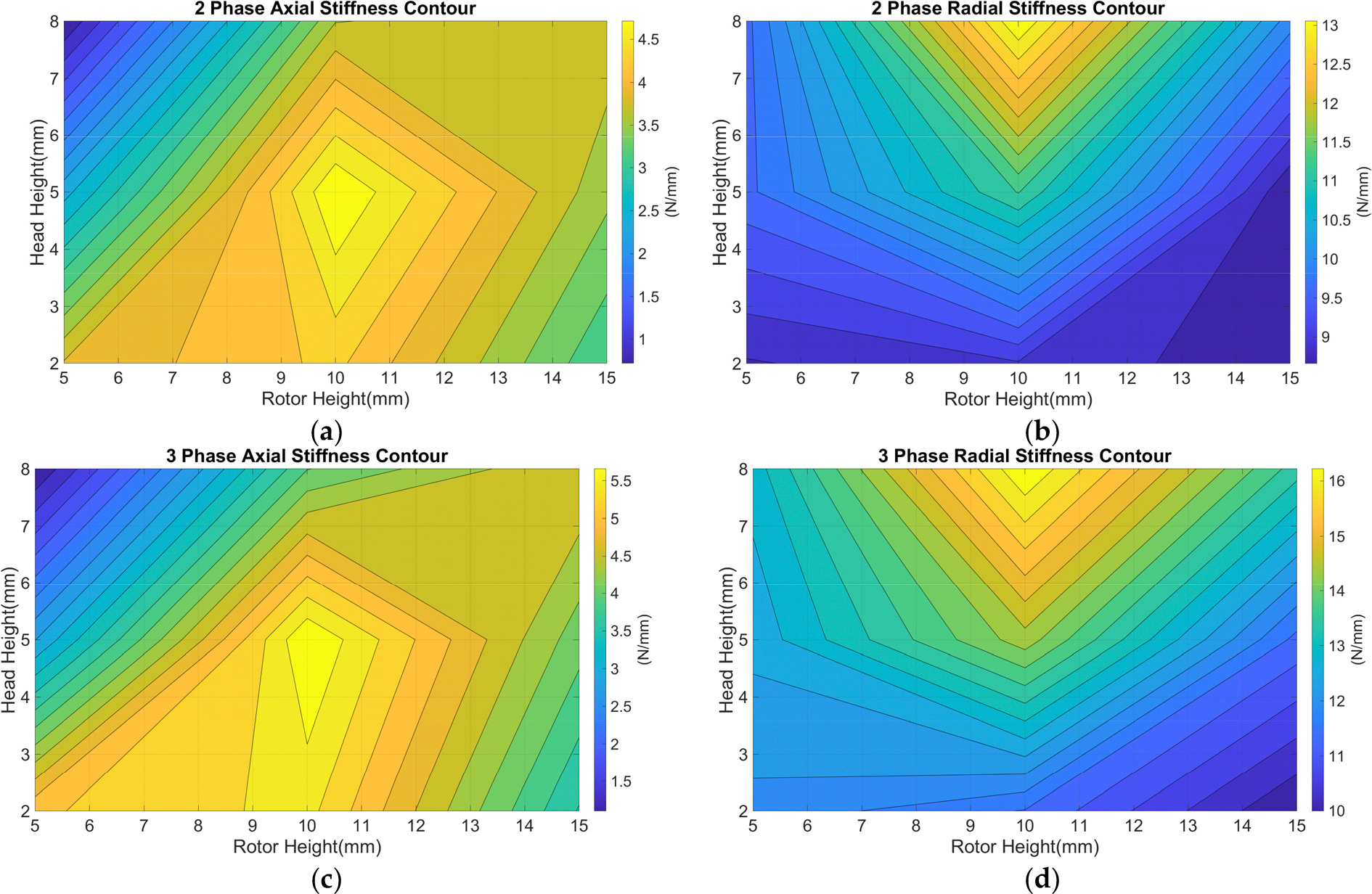
The relationship between rotor height and head height. (**a**) The two-phase axial stiffness contour, (**b**) the two-phase radial stiffness contour, (**c**) the three-phase axial stiffness contour, and (**d**) the three-phase radial stiffness contour. Note that, because the current force was monotonic for these geometric combinations, it was omitted here.

There are two geometric features whose sensitivities are non-linear and, thus, excluded from Figure 8: the head height and rotor height. These sensitivities also exhibit non-monotonic behavior, and this suggests an interdependent relationship between them. As mentioned previously, the relationship between the two geometric features was explored by conducting simulations for the six additional combinations of the head height and rotor height. The results of these studies are seen in Figure 9.

Figure 9 demonstrates the interdependent relationship between the two geometric features. We found that the axial stiffness is maximized when the dimensions of the rotor height and head height are 10 mm and 5 mm, respectively. This indicates that a rotor-height-to-head-height ratio of approximately 2:1 may be desirable for maximizing the axial stiffness. Conversely, we did not observe a similar trend for the radial stiffness because its visible maximum is when the dimensions of the rotor height and head height are 10 mm and 8 mm, respectively. These trends suggest that the combination of the rotor height and head height for maximum radial stiffnesses lay outside of the dimensions simulated in this study. To speculate, the combination of the two geometric features which results in the maximum radial stiffness is likely 10 mm and 10 mm. This would then suggest that a 1:1 ratio maximizes the radial stiffness.

## 4. Conclusions

In this study, two-phase and three-phase bearingless permanent magnet slice motor configurations were compared. Initially, two nominal designs were manufactured, and their performance characteristics were empirically tested. The three characteristics of the axial stiffness, radial stiffness, and current force were used as the metrics by which to evaluate these nominal designs. FEA models were created based upon the nominal devices and were validated by the empirical results. Consequently, simulations were used to vary seven different geometric features of the model to determine the sensitivity of the two-phase and three-phase configurations. Our findings showed that the nominal three-phase had a higher passive axial stiffness, but that was accompanied by a higher radial stiffness, which resulted in a modestly (6.4%) lower axial-to-radial-stiffness ratio. This is a meager advantage for the two-phase design. Furthermore, we showed that the nominal two-phase design has a higher (45.4%) current force, but, when considering the force generated as a function of power, the nominal three-phase design was slightly superior (2.7%). Bear in mind the three-phase nominal design would require more power to counteract the radial forces due to its higher radial stiffness.

To determine which configuration to use for a blood pump, designers would have to consider aspects such as the size, air gap, and prevalence of off-the-shelf controller electronics for each configuration. As mentioned previously, size is a major consideration when designing an implantable blood pump as there is the desire for them to be compact. One of the limitations as to how small a BPMSM blood pump can be made relates to the fact that BPMSMs require space between the stator arms for parts such as coils and sensors. With that said, the three-phase configuration presented in this study has more stator arms than its two-phase counterpart. While having more stator arms does not inherently impact the performance of a three-phase device, it does limit the extent to which it can be scaled down relative to a comparable two-phase device. This is simply because a three-phase device would have less room between the arms while maintaining adequate performance characteristics. It is, then, in this regard that the two-phase configuration presented here would have the advantage for usage in an implantable blood pump.

For the air gap, it was shown that the performance characteristics of both configurations are highly sensitive to even minor changes in the dimension. The three-phase configuration was shown to have a higher sensitivity to air gap changes, but this stems from its larger surface area compared to the two-phase design [28]. Furthermore, it is important to note that the air gap itself does not directly affect this surface area. Consequently, neither the two-phase nor three-phase configuration would inherently be better equipped to handle a larger air gap than the other.

The last thing to consider is the availability of off-the-shelf components because these can save on both development and manufacturing costs. In general, traditional electric three-phase motors are much more established in the modern era than two-phase motors [32]. As a result of this, the research and development of electric motors and their peripherals are predominantly centered around the three-phase configuration. This results in a myriad of off-the-shelf robust motor drive controllers which can control the rotational portion of the device that only work with three-phase configurations [25]. This becomes the only major drawback of the two-phase configuration as there is the need to develop a bespoke two-phase motor drive controller. Overall, the two-phase configuration’s benefits outweigh its drawbacks for the application of an implantable blood pump within the context of this study.

Regardless of configuration, both showed consistent trends in their sensitivity to dimensional changes in specific geometric features. We determined that changes in the air gap and rotor length lead to the most drastic deviations in the three performance characteristics. Conversely, the rotor outer radius provided a negligible effect on the performance characteristics. While the majority of changes in the geometric features contained tradeoffs, increasing the head protrusion only benefited the overall performance. Furthermore, we elucidated the interdependent nature of the geometric features of the head height and rotor height. This may prove extremely useful to future motor designers.

## Supplementary Material

Supplementary Material Lawley 2024

**Supplementary Materials:** The following are available online at https://www.mdpi.com/article/10.3390/act13050179/s1, Figure S1: (a) A cut side view of the rotor and stator where the *ϕ* denotes the tilt angle. The black dot denotes the axis of about which the rotor tilts. (**b**) A top view of the rotor and stator where the dotted line indicates the axis of tilt; Figure S2: The numerical results for the rotor torque as a function of tilt angle for the two-phase nominal design. The slope of Figure S2 is the tilting stiffness which in this case is 0.0339 (Nm/°). thus demonstrating the equation’s ability to approximate the tilting stiffness based upon axial stiffness; Figure S3: A general schematic of the connections of the various components of the force testing rig. An arrow between blocks indicate signal direction as well as the fact that a physical connection and electrical connection exists between these two components. Solid lines between blocks indicate that only a physical connection exists between the two components. The dotted line demonstrates the magnetic coupling between the rotor and stator and thus no physical or electrical connection; Figure S4: Comparison of numerical and empirical data for axial stiffness, radial stiffness, and current force of the nominal design. (a) The two-phase nominal design and (b) the three-phase nominal design; Figure S5: The sensitivity of the following performance characteristics as a function of the geometric features, (a) axial stiffness, (b) radial stiffness, (c) current force, and (d) axial to radial stiffness; Table S1: The sensitivity values for each performance characteristic. Additionally the percent difference for each performance characteristic between the two-phase and three-phase configurations. * The value for this position is much smaller than the rest with it only being 0.00009 (N/mm^2^).

## Figures and Tables

**Table 1.** The key geometric features evaluated in the simulations. The bolded numbers indicate the dimensions for the nominal designs. All dimensions are in millimeters.

Air Gap	Head Height	Head Protrusion	Head Width	Rotor Height	Rotor Length	Rotor Outer Radius

1.5	2	1	4	5	**3.5**	**26**
**2**	**5**	**3**	6	**10**	5.5	29
2.5	8	5	**8**	15	7.5	32

**Table 2.** Comparison between numerical and empirical data.

Characteristics	Two-Phase Numerical	Two-Phase Empirical	Two-Phase Percent Difference	Three-Phase Numerical	Three-Phase Empirical	Three-Phase Percent Difference

Axial Stiffness (N/mm)	4.15	4.60	10.3	5.43	6.08	11.3
Radial Stiffness (N/mm)	10.60	13.44	20.4	14.05	17.34	21.0
Current Force (N/A)	1.97	2.26	13.5	1.24	1.49	18.1

**Table 3.** Performance characteristics of the two-phase and three-phase nominal designs.

Characteristics	Two-Phase	Three-Phase	Percent Difference

Axial Stiffness (N/mm)	4.15	5.43	26.7
Radial Stiffness (N/mm)	10.60	14.05	27.9
Current Force (N/A)	1.97	1.24	45.4
Axial-to-Radial Stiffness	0.392	0.386	6.4

## Data Availability

The data presented in this study are available upon request from the corresponding author.
