# Supplementary material for: Practical Comparison of Two- and Three-Phase Bearingless Permanent Magnet Slice Motors for Blood Pumps
Source: Actuators. Author manuscript; Available in PMC 2026 Jun 18. (PMC13274715; doi:10.3390/act13050179)
Supplement: Supplementary Material Lawley 2024 [file NIHMS2154062-supplement-Supplementary_Material_Lawley_2024.pdf]

Supplemental Material

Supplemental Section S1.

Due to the fact that the ratio of rotor diameter to rotor height is high in all BPMSMs, then small ϕ values result primarily in the axial displacement of the rotor. The relationship between axial displacement and axial force was well characterized in this study and is used here to approximate its tilting stiffness. Tilting stiffness is the restoring torque resulting from the rotor being tilted about either the x or y axis and is defined by

$$Tilt_{stiffness} = \frac{T}{\phi} \tag{1}$$

, where T is the restoring torque (Nm) and ϕ is the deflection angle (°). Figure S1 demonstrates the coordinate system used for this derivation.

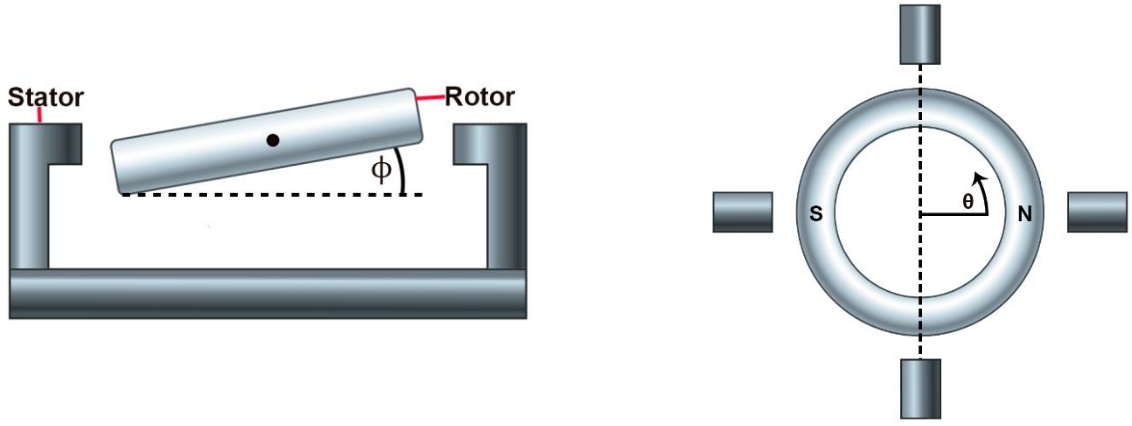

(**a**) (**b**)

Figure S1: (**a**) A cut side view of the rotor and stator where the ϕ denotes the tilt angle. The black dot denotes the axis of about which the rotor tilts. (**b**) A top view of the rotor and stator where the dotted line indicates the axis of tilt.

Torque is defined as

$$T = F \cdot D \tag{2}$$

where F is force (N), and D is the distance from the axis of rotation (m). In the case of a tilting rotor, where both F and D vary as a function of theta, T can be found by integrating equation 2 around the rotor, as follows

$$T = \int_0^{2\pi} F\ D\ d\theta \tag{3}$$

Here, θ denotes the angular position around the rotor. The moment arm, D, around the rotor is defined as

$$D(\theta) = R\ |\cos(\theta)| \tag{4}$$

, where R is radius of the rotor (m).

The magnetic force between a permanent magnet and a ferrous object is dependent on the scalar magnitude of magnetic flux density and is always attractive. The rotor utilized in our study is a diametrically magnetized dipole magnet, resulting in a sinusoidal flux pattern. Consequently, forces follow a similar pattern. We can then represent the distribution of force on the rotor as follows,

$$F(\theta) = F_0|\cos(\theta)| \tag{5}$$

, F_0 is a scaling factor(N) that needs to be solved. Here cosine is used because the θ angle of 0° corresponds with a point on the rotor with the largest force based on our established coordinate system and rotor orientation. It can be shown that for rotor axial displacements F_0 is defined as

$$F_0(Z) = \frac{Ax_s\, Z}{4} \tag{6}$$

, where Ax_s is the axial stiffness (N/m), Z is the axial distance of the rotor(m) from the neutral position.

When we consider that when the rotor is tilted by some angle ϕ, then Z varies around the rotor based upon the angular positions, θ. Z can then be represented as follows

$$Z(\theta, \phi) = R\sin(\phi)\,|\cos(\theta)| \tag{7}$$

By placing equations 6 and 7 into equation 5 we then get

$$F(\theta) = \frac{Ax_s\, R\ \sin(\phi)\,|\cos^2(\theta)|}{4} \tag{8}$$

Then substituting equation 4 and 8 into equation 2

$$T = \int_0^{2\pi} \frac{Ax_s R^2\ \sin(\phi)\,|\cos^3(\theta)|}{4}\,d\theta \tag{9}$$

As a result of both force and distance being symmetric about the tilt axis then we can simplify equation 9 to

$$T = \int_{-\pi/2}^{\pi/2} \frac{Ax_s R^2\ \sin(\phi)\cos^3(\theta)}{2}\,d\theta \tag{10}$$

and is equivalent to

$$T = \frac{2Ax_s R^2 \sin(\phi)}{3} \tag{11}$$

This expression can be evaluated for an angle of 1 degree to evaluate the tilting stiffness.

$$Tilt_{stiffness} = \frac{T}{1°} = \frac{2\ Ax\ R^2 \sin(1°)}{3} \tag{12}$$

For our bearing, the axial stiffness is 4.15 N/mm (4150 N/m) and R= 0.026m, the calculated tilting stiffness is 0.0326(Nm/°),

Supplemental Section S2.

To compare the derived equation we simulated the tilting stiffness for the nominal 2-phase design. For this simulation we displaced the rotor in our simulation evaluated the torque for tilt angles from -4 to 4 in 1 degree increments, the data of this is shown in figure S2.

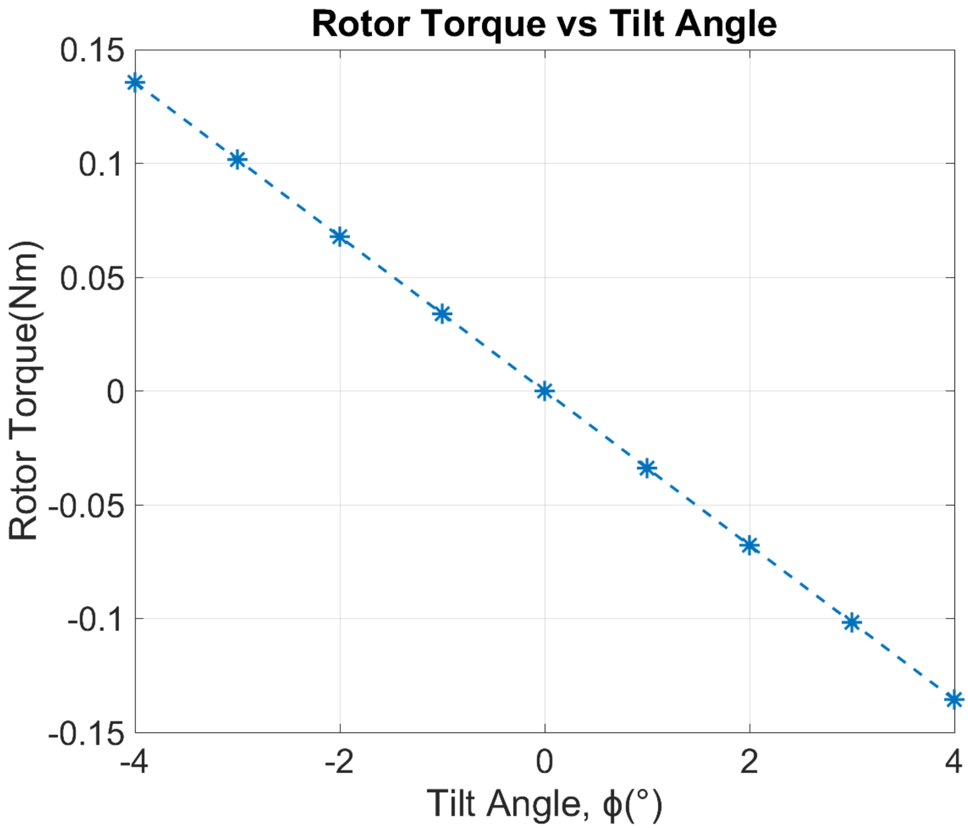

Figure S2: The numerical results for the rotor torque as a function of tilt angle for the 2-phase nominal design.

The slope of figure S2 is the tilting stiffness which in this case is 0.0339 (Nm/°). thus demonstrating the equation's ability to approximate the tilting stiffness based upon axial stiffness.

Supplemental Section S3

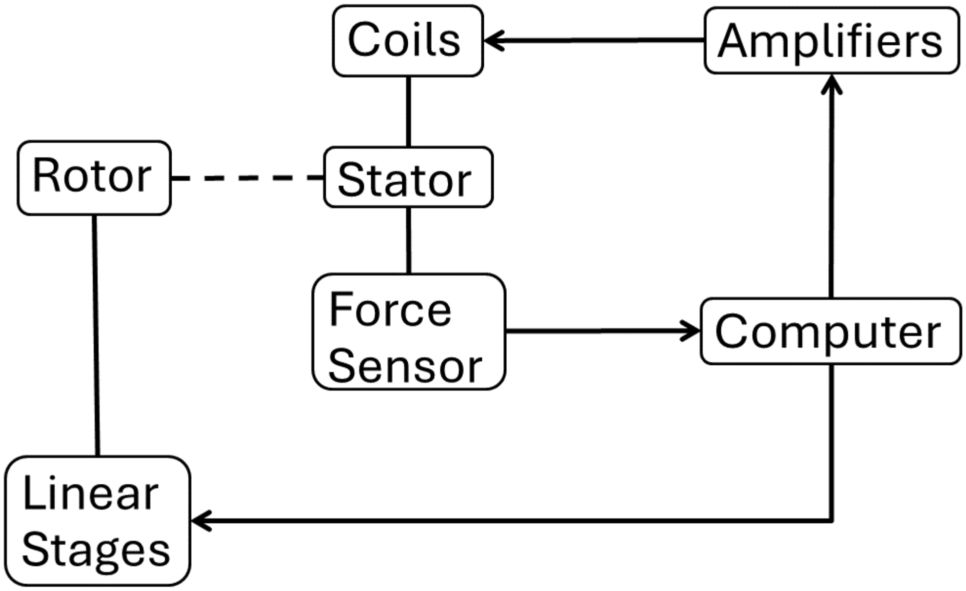

Figure S3: A general schematic of the connections of the various components of the force testing rig. An arrow between blocks indicate signal direction as well as the fact that a physical connection and electrical connection exists between these two components. Solid lines between blocks indicate that only a physical connection exists between the two components. The dotted line demonstrates the magnetic coupling between the rotor and stator and thus no physical or electrical connection.

Supplemental Section S4

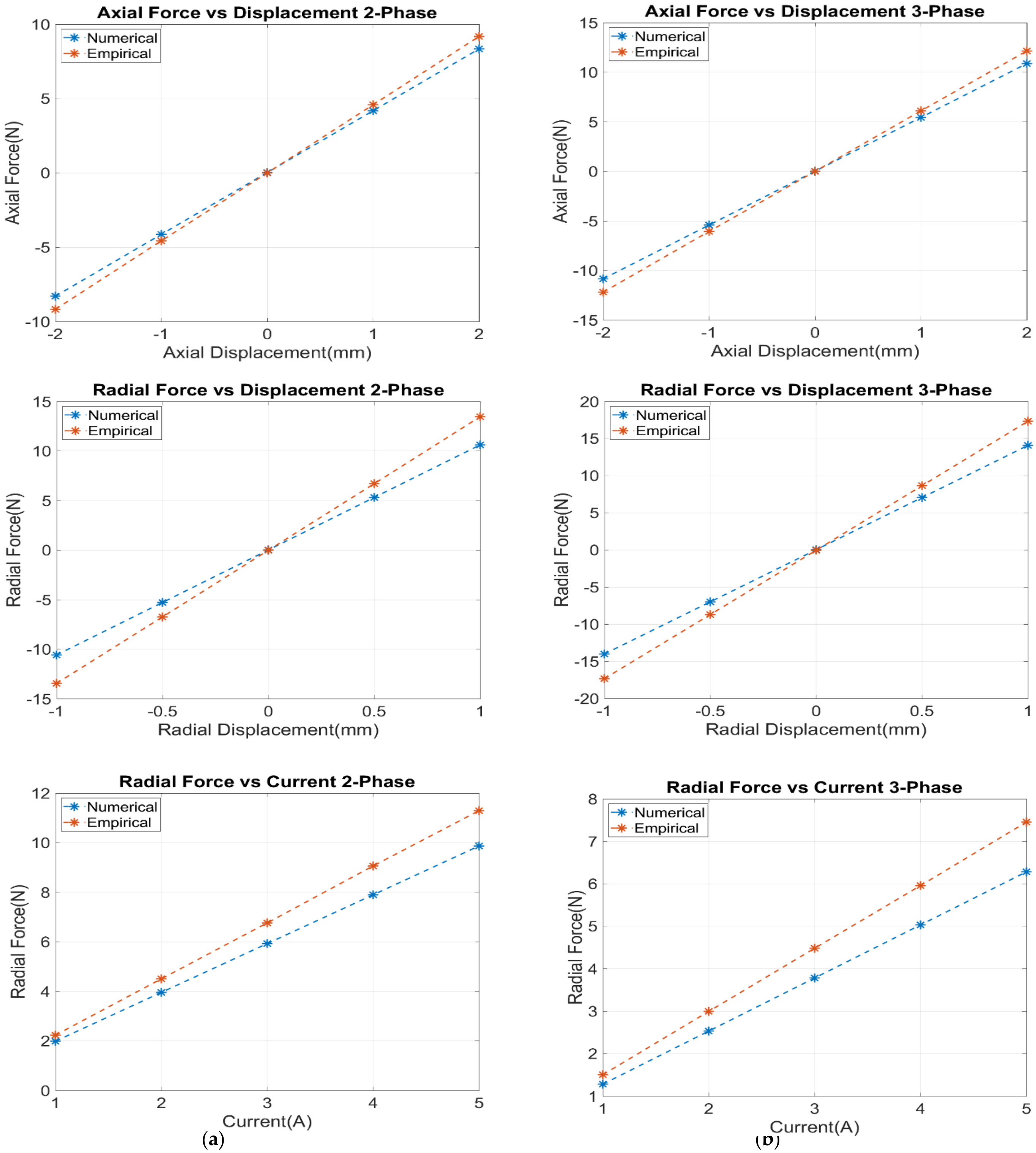

Figure S4: Comparison of numerical and empirical data for axial stiffness, radial stiffness, and current force of the nominal design. (**a**) The 2-phase nominal design and (**b**) the 3-phase nominal design.

Supplemental Section S5.

Table S1: The sensitivity values for each performance characteristic. Additionally the percent difference for each performance characteristic between the 2-phase and 3-phase configurations. * The value for this position is much smaller than the rest with it only being 0.00009 (N/mm^2).

Geometric Feature	2-Phase Axial Stiffness (N/mm^2)	2-Phase Radial Stiffness (N/mm^2)	2-Phase Current Force ($N\,A/mm^2$)	2-Phase Axial to Radial Stiffness ($1/mm$)	3-Phase Axial Stiffness (N/mm^2)	3-Phase Radial Stiffness (N/mm^2)	3-Phase Current Force ($N\,A/mm^2$)	3-Phase Axial to Radial Stiffness ($1/mm$)
Air gap	-1.883	-3.899	-0.298	-0.021	-2.336	-5.185	-0.168	-0.014
Head Protrusion	0.465	-0.198	0.012	0.049	0.577	-0.201	0.0064	-0.043
Head Width	0.277	0.854	0.046	-0.012	0.312	0.991	0.0053	-0.008
Rotor Length	1.891	3.977	0.525	0.007	2.338	5.365	0.211	0.004
Rotor Outer Radius	0.055	-0.026	-0.037	0.005	0.012	~0*	-0.011	0.0006

Geometric Feature	Axial Stiffness Percent Difference	Radial Stiffness Percent Difference	Current Force Percent Difference	Axial to Radial Stiffness Percent Difference
Air gap	21.2	28.3	59.8	40.6
Head Protrusion	21.6	6.1	76.1	3.6
Head Width	13.4	15.2	153.4	35.3
Rotor Length	20.8	29.8	85.1	53.9
Rotor Outer Radius	137.9	201.8	105.8	158.1

Supplemental Section S6.

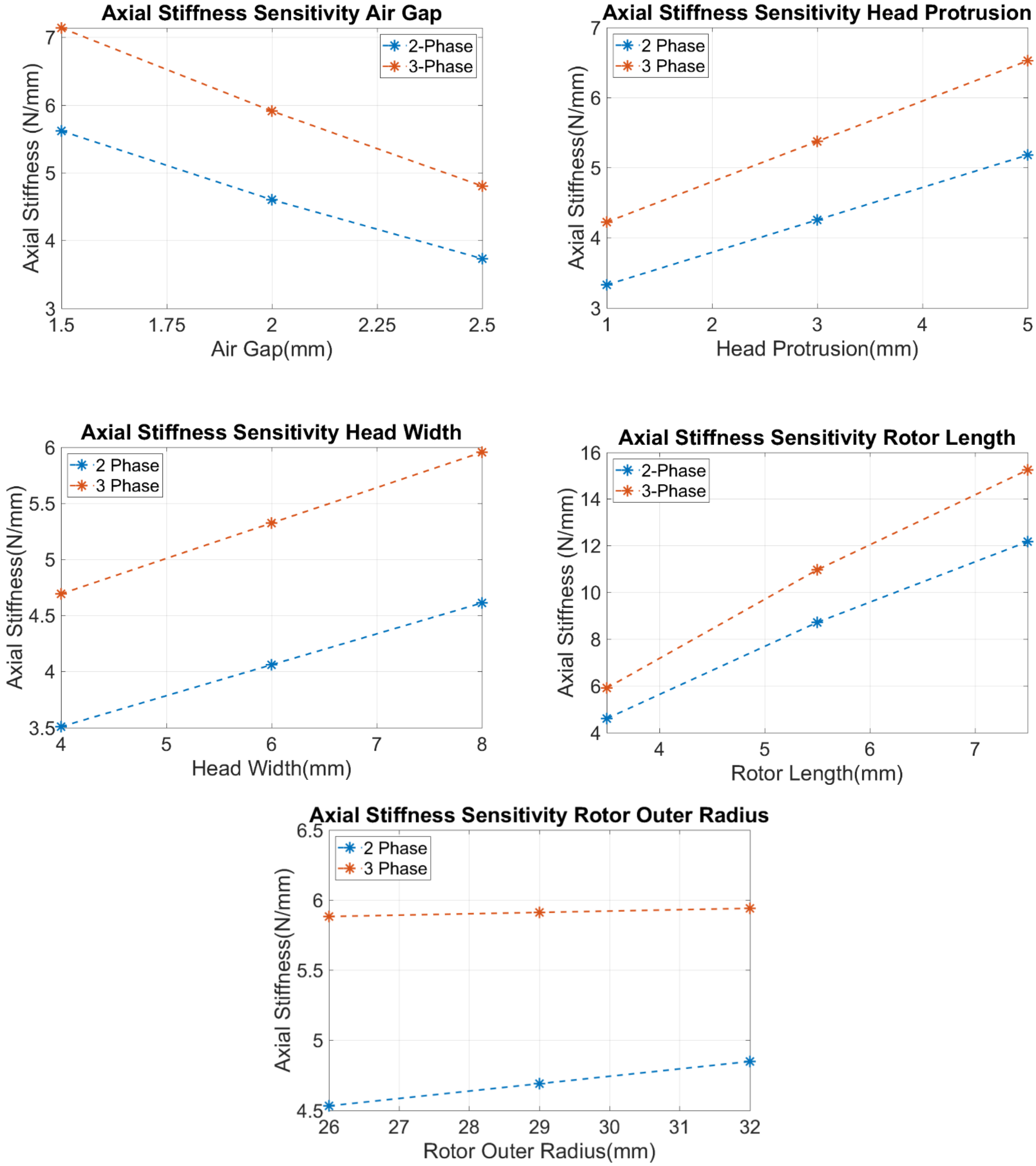

(**a**)

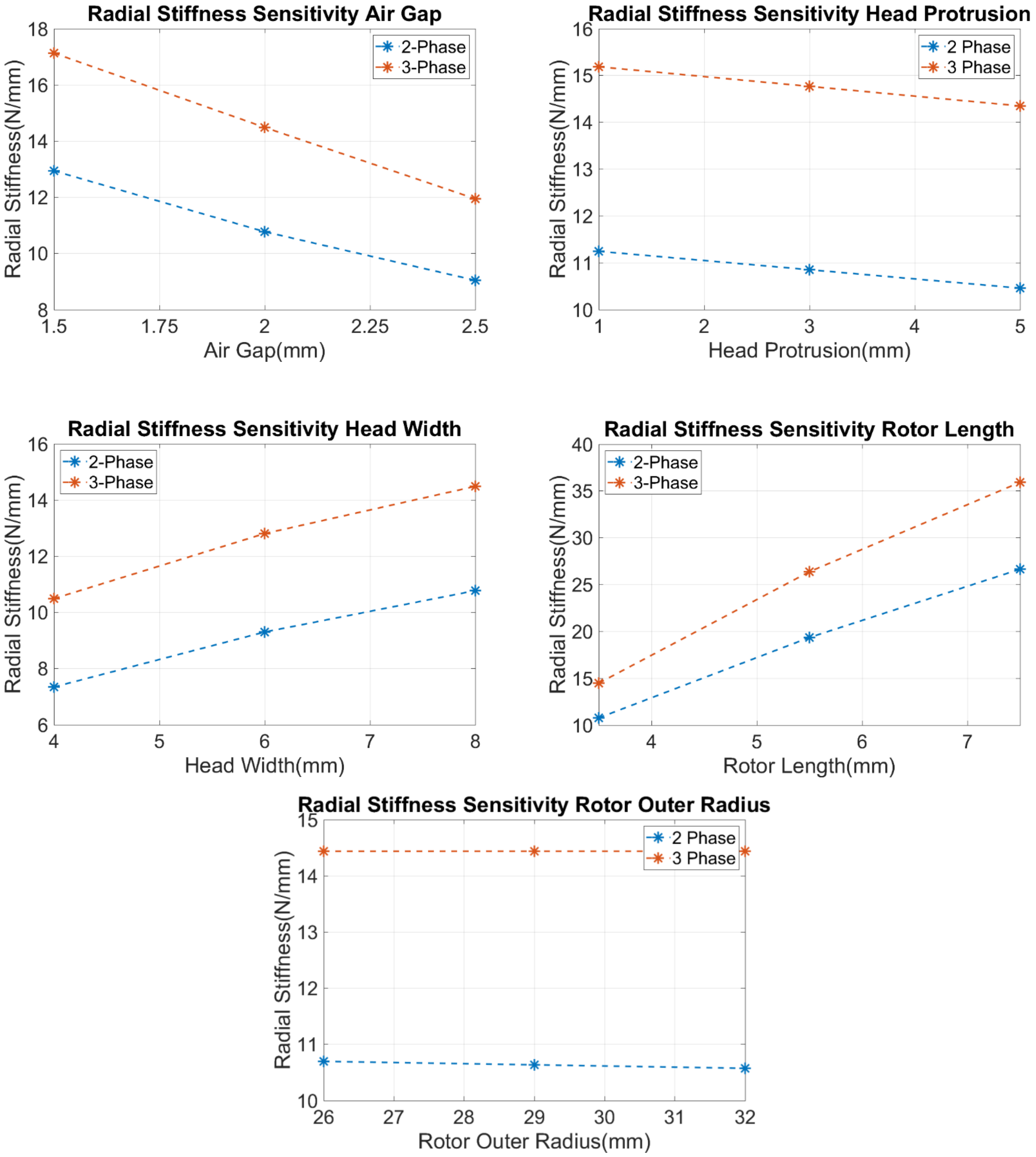

Radial Stiffness Sensitivity Air Gap
Radial Stiffness(N/mm)
Air Gap(mm)
2-Phase
3-Phase
18
16
14
12
10
8
1.5
1.75
2
2.25
2.5
Radial Stiffness Sensitivity Head Protrusion
Radial Stiffness(N/mm)
Head Protrusion(mm)
2 Phase
3 Phase
16
15
14
13
12
11
10
1
2
3
4
5
Radial Stiffness Sensitivity Head Width
Radial Stiffness(N/mm)
Head Width(mm)
2-Phase
3-Phase
16
14
12
10
8
6
4
5
6
7
8
Radial Stiffness Sensitivity Rotor Length
Radial Stiffness(N/mm)
Rotor Length(mm)
2-Phase
3-Phase
40
35
30
25
20
15
10
4
5
6
7
Radial Stiffness Sensitivity Rotor Outer Radius
Radial Stiffness(N/mm)
Rotor Outer Radius(mm)
2 Phase
3 Phase
15
14
13
12
11
10
26
27
28
29
30
31
32

(**b**)

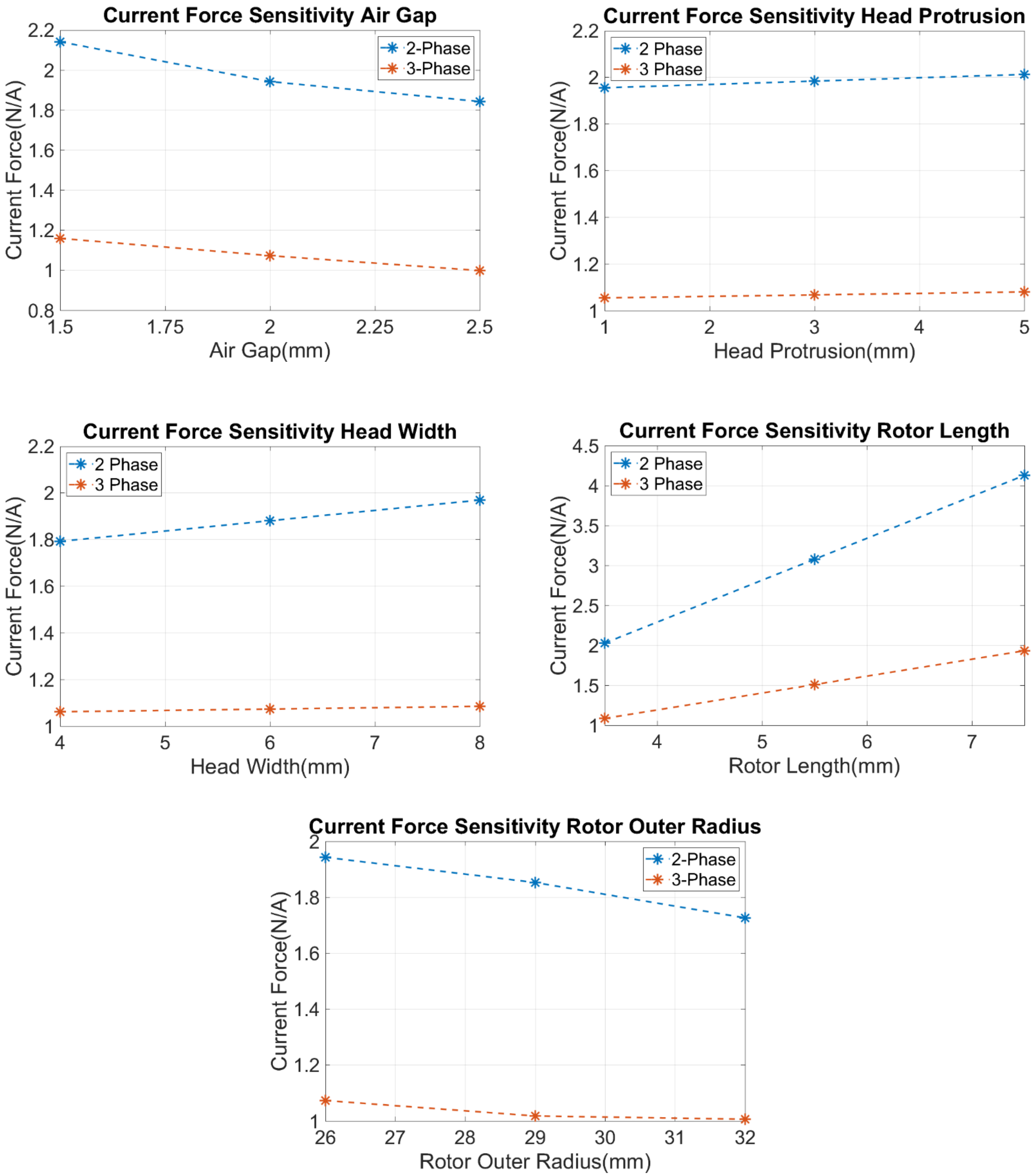
Current Force Sensitivity Air Gap
2-Phase
3-Phase
Current Force(N/A)
Air Gap(mm)
Current Force Sensitivity Head Protrusion
2 Phase
3 Phase
Current Force(N/A)
Head Protrusion(mm)
Current Force Sensitivity Head Width
2 Phase
3 Phase
Current Force(N/A)
Head Width(mm)
Current Force Sensitivity Rotor Length
2 Phase
3 Phase
Current Force(N/A)
Rotor Length(mm)
Current Force Sensitivity Rotor Outer Radius
2-Phase
3-Phase
Current Force(N/A)
Rotor Outer Radius(mm)

(**c**)

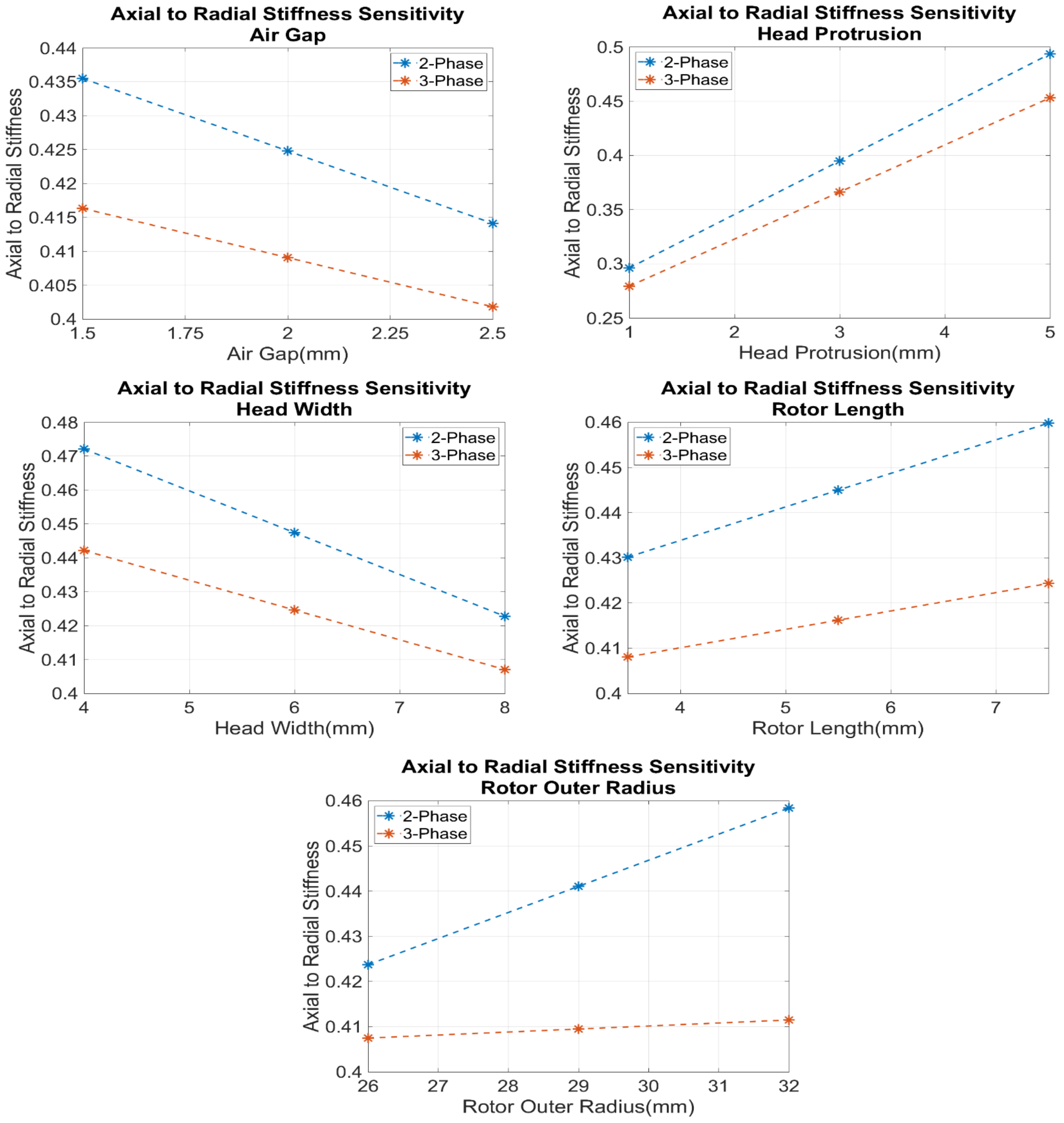

(**d**)

Figure S5: The sensitivity of the following performance characteristics as a function of the geometric features, (**a**) axial stiffness, (**b**) radial stiffness, (**c**) current force, and (**d**) axial to radial stiffness.
